# Gastroduodenal Outlet Obstruction and Palliative Self-Expandable Metal Stenting: A Dual-Centre Experience

**DOI:** 10.1155/2013/167851

**Published:** 2013-11-10

**Authors:** Nik S. Ding, Sina Alexander, Michael P. Swan, Christopher Hair, Patrick Wilson, Emma Clarebrough, David Devonshire

**Affiliations:** ^1^Endoscopy Unit, Barwon Health, Geelong, VIC 3220, Australia; ^2^School of Medicine, Deakin University, Geelong, VIC 3220, Australia; ^3^Endoscopy Unit, Monash Health, Clayton, VIC 3168, Australia

## Abstract

*Background*. Self-expandable metal stents (SEMs) are increasingly being utilised instead of invasive surgery for the palliation of patients with malignant gastroduodenal outlet obstruction. *Aim*. To review two tertiary centres' experience with placement of SEMs and clinical outcomes. *Methods*. Retrospective analysis of prospectively collected data over 12 years. *Results*. Ninety-four patients (mean age, 68; range 28–93 years) underwent enteral stenting during this period. The primary tumour was gastric adenocarcinoma in 27 (29%) patients, pancreatic adenocarcinoma in 45 (48%), primary duodenal adenocarcinoma in 8 (9%), and cholangiocarcinoma and other metastatic cancers in 14 (16%). A stent was successfully deployed in 95% of cases. There was an improvement in gastric outlet obstruction score (GOOS) in 84 (90%) of patients with the ability to tolerate an enteral diet. Median survival was 4.25 months (range 0–49) without any significant differences between types of primary malignancy. Mean hospital stay was 3 days (range 1–20). Reintervention rate for stent related complications was 5%. *Conclusion*. The successful deployment of enteral stents achieves excellent palliation often resulting in the prompt reintroduction of enteral diet and early hospital discharge with minimal complications and reintervention.

## 1. Introduction

Malignant gastroduodenal obstruction is a late and severe complication that develops in up to 20% of patients with advanced carcinoma of the pancreas, stomach, or the duodenum [1–3]. Patients may present with nausea, vomiting, and weight loss with resultant impairment in quality of life [4]. Palliative interventional procedures, either surgical or endoscopic, offer a rapid nonpharmacological modality to improve symptoms as measured by the gastric outlet obstruction score (GOOS) [5].

Although surgery for established gastric outlet obstruction is technically successful in up to 90% of patients [6], it is often associated with a prolonged hospital stay and sometimes with poor function of gastroenterostomy [7].

 Curative surgical resection is often not possible and palliative surgical bypass operations have been associated with high mortality and morbidity rates of up to 30% and 50%, respectively [8–10]. Even with the improvements in surgical care and laparoscopic techniques, the more recent reported rates of mortality and morbidity are 10% and 30%, respectively [11–13]. 

Self-expandable metal stents (SEMs) are devices that are used in the alimentary tract to help alleviate symptoms caused by oesophageal, gastroduodenal, biliary, and colonic malignancies [14, 15]. Endoscopic stent deployment for gastroduodenal obstruction, often performed under sedation, has been shown to be a safe alternative to surgical bypass. Up to 92% of patients can consume an enteral diet and up to 73% can tolerate solid or semisolid food following stent deployment [16].

Endoscopic therapy has the advantages of being a well-tolerated day stay or short stay procedure associated with a low complication rate and rapid symptom relief. It is effective in the majority of cases and often no further reintervention is necessary. 

In this large dual-centre study, we report on our technical success and the clinical outcomes of patients with gastroduodenal outlet obstruction treated with SEMs.

## 2. Methods

A retrospective, nonrandomised study was performed using prospectively collected data in two tertiary care hospitals in Australia over a 12-year period. Patients over 18 years with symptomatic gastroduodenal obstruction who were not surgical candidates were included in the study. Patients with multiple lesions, intestinal ischaemia, and contraindication to gastrointestinal endoscopy were excluded. 

All patients had been considered unsuitable for surgical intervention prior to referral and were unable to tolerate enteral nutrition at the time of referral. Without intervention, these patients would have required a nasogastric tube or venting gastrostomy to relieve obstruction. The diagnosis of obstruction was confirmed by endoscopy or barium studies prior to intervention. Patients with biochemical evidence of contemporaneous biliary obstruction underwent endoscopic (or, if not feasible, radiological) placement of a metal biliary stent prior to gastroduodenal stent insertion. All patients gave informed consent for the intervention. 

We defined technical success as successful endoscopic and fluoroscopic placement of stent. Clinical success was defined as time to resumption and/or improvement of oral intake (defined by gastric outlet obstruction scoring system (GOOSS) score, with 0 = no oral intake, 1 = liquids only, 2 = soft foods, and 3 = solid food/full diet) and duration of patient survival. This information was collected after reviewing patient's clinical history. 

Patients were discharged when able to tolerate at least a liquid/softened diet. Follow-up data were obtained by reviewing the medical records and by contacting the referring physician or the patient's general practitioner. Information obtained included the occurrence of complications and the need for reintervention, the type of diet that was tolerated, and the duration of survival.

### 2.1. Technique of Stent Insertion

**Figure 1:**
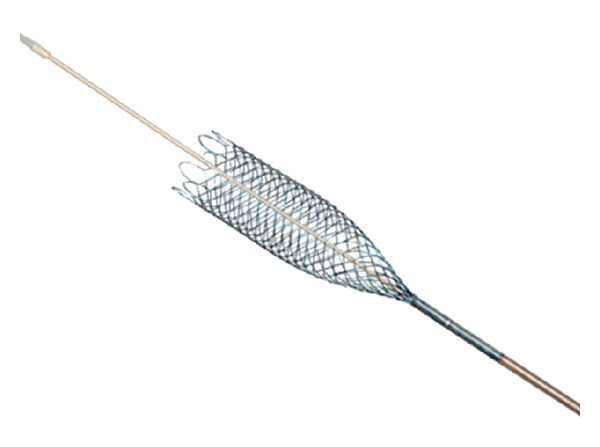
Self-expandable metal stent (courtesy of Boston Scientific Corporation).

SEMs are packaged in a compressed form for delivery and consist of various alloy mesh cylinders. They are available in various lengths and diameters. Once deployed, they are designed to exert self-expansive forces until they reach their maximum fixed diameter (Figure 1). To prevent migration, most SEMs have a proximal and/or distal flare.

During the first 7 years of our study, 60 or 90 mm long, 20 or 22 mm long outer diameters through the scope Wallstents (Boston Scientific Corporation, MA, USA) were used. During the last 5 years, predominantly newer WallFlex stents (60, 90, or 120 mm long, 22 mm body) were used (Boston Scientific Corporation, MA, USA). All stents were uncovered. 

**Figure 2:**
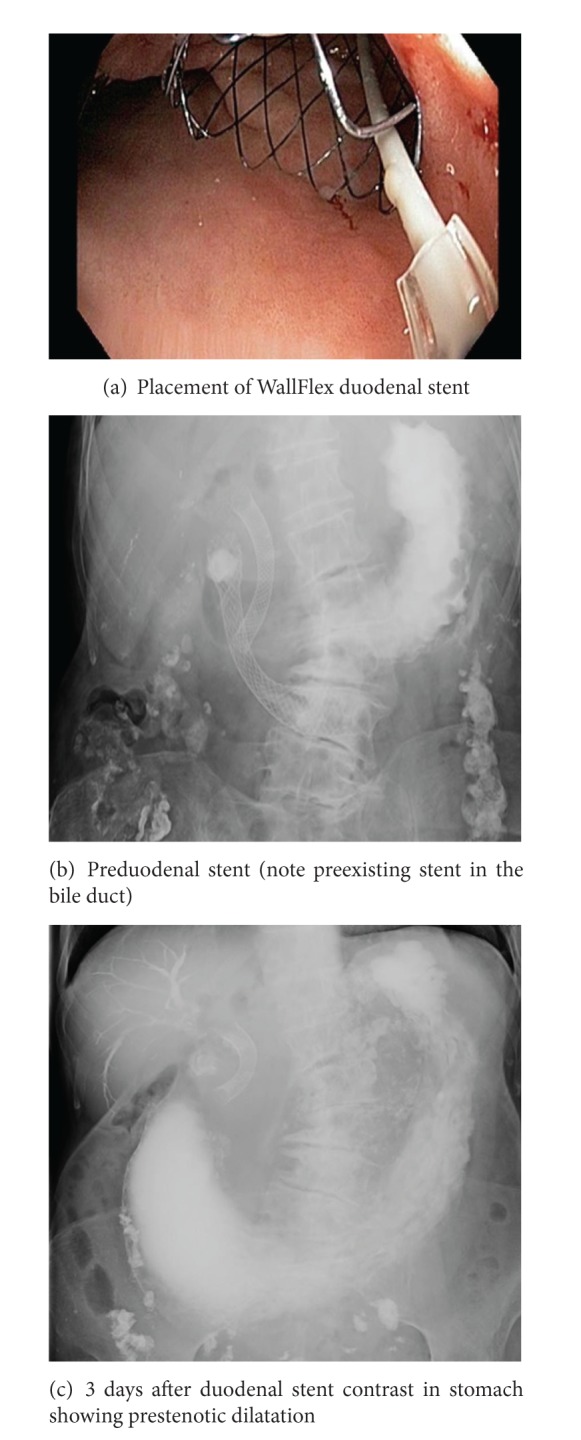
Fluoroscopic and endoscopic view of deployed stent.

Stent placement was conducted under sedation or general anaesthesia and the identification of the structure was required endoscopically. A 0.035-inch (0.9 mm) guidewire was subsequently used to traverse the stricture under fluoroscopic guidance. The stent was then positioned across the stricture and deployed (Figure 2). The length of the stent used was determined by the endoscopist at the time of the procedure based upon the length of the stricture and the position of the distal and proximal ends of the stent in the anatomical shape of the duodenum. Contrast was injected immediately before and after stent insertion to estimate the tumour length and to confirm that the guidewire was within the small bowel lumen.

## 3. Results and Discussion

Between January 2000 and June 2012, 94 patients underwent enteral stent placement for malignant gastroduodenal obstruction. There were 51 females and 43 males with a mean age of 68 years (range 34–93 years) (Table 1). All patients had been deemed unsuitable for surgical gastroenterostomy prior to referral. 75% required a nasogastric tube at presentation for suction and symptomatic relief, indicating advanced disease.

**Figure 3:**
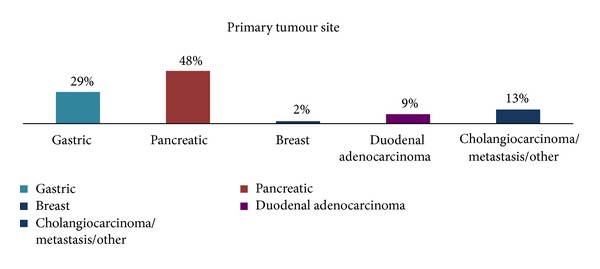
Graphical representation of underlying primary malignancy.

The primary diagnosis was gastric adenocarcinoma in 27 (29%) patients, pancreatic adenocarcinoma in 45 (48%), primary duodenal adenocarcinoma in 8 (9%), and cholangiocarcinoma and other metastatic diseases in 14 (16%) (Figure 3). The stent position was duodenal in 44 patients (47%), gastric in 40 (43%), and jejunal in 10 patients (10%).

**Figure 4:**
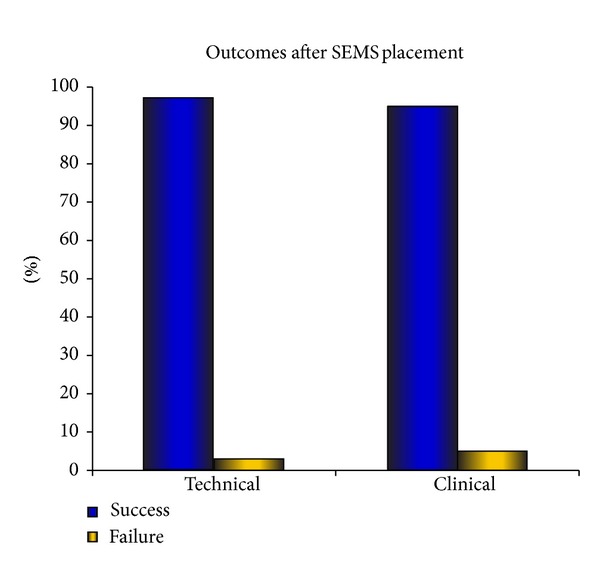
Graph showing clinical and technical success of stent placement.

Enteral stent placement was technically successful in 89 (95%) patients and clinically successful in 84 (90%) with all of these showing improvement in gastric outlet obstruction score (GOOS) (Figure 4). There was a one-point improvement of GOOS in these patients. Fifty-six (60%) patients had Wallstents inserted; thirty-three (35%) had WallFlex stents; and four (4%) patients had both. 

**Figure 5:**
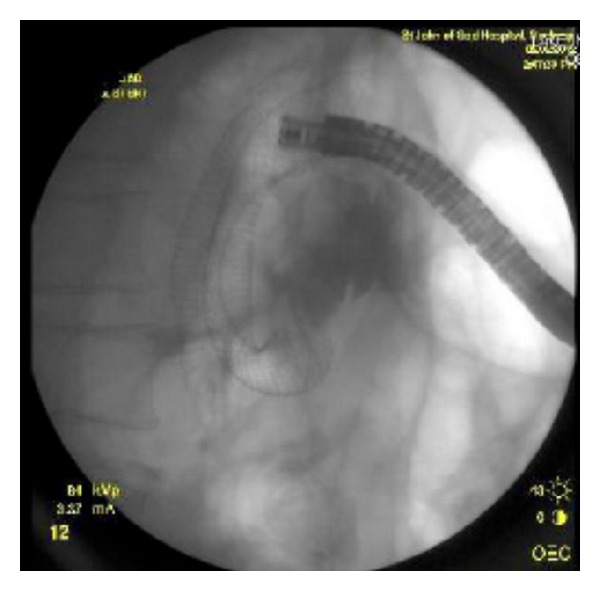
Tumour ingrowth treated with insertion of a second stent.

The average length of stay was 3 hospital days (range 1–20). In 5 cases (5%), stent reinsertion was undertaken due to tumour ingrowth (Figure 5). This was seen in three patients with pancreatic adenocarcinoma and two with duodenal adenocarcinoma. These additional stents were all successfully deployed with an average time to restent of 3 months (range 1–5). No stent migration was noted.

Complications encountered were perforation in one patient and aspiration pneumonia in 5 patients. The perforation occurred in a 90-year-old patient with subsequent death; all the aspiration cases required prolonged hospital admission and administration of intravenous antibiotics. 

**Figure 6:**
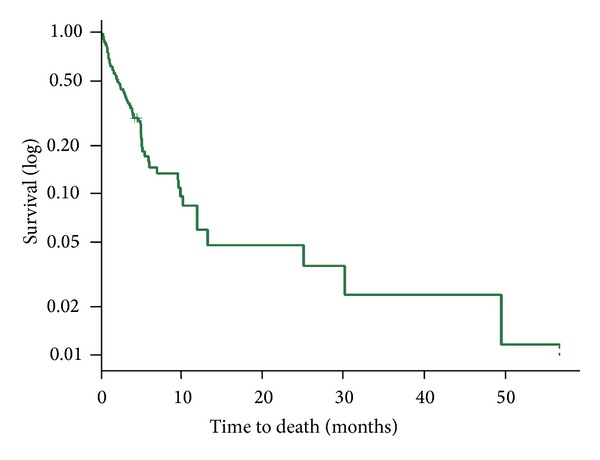
Kaplan-Meier plot of patient survival after stent insertion.

The average survival after stent placement was 4.25 months with a median survival of 2 months (0–49 months) (Figure 6). There were no significant differences in survival between patients with gastric or pancreatic cancers, with median survival of 2 months range (0.5–49) (Table 2). 

Thirty-one patients (32.9%) survived less than one month after stent placement. The cause of death in this group was from metastatic disease and did not relate to stent failure from tumour ingrowth. 

Following stent insertion, 84 (90%) of patients were able to recommence oral intake (either solids or liquids). In ten patients, no enteral feeding could be commenced. There was notably median survival of 2 weeks in this group.

## 4. Discussion

Patients with malignant gastroduodenal obstruction often have a limited life expectancy and will rapidly deteriorate from complications relating to obstructive symptoms and starvation [2]. Many of these patients are not surgical candidates due to poor nutrition and general health [3]. A surgical gastroenterostomy has a high success rate in bypassing their obstruction, but it is associated with a morbidity of up to 40% and occasional mortality [13, 17, 18] whilst extending the hospital stay by at least 2 weeks [6].

This study demonstrates that in patients with malignant gastroduodenal obstruction who are unsuitable for surgery, endoscopic stent placement can result in rapid resolution of symptoms (reduction of GOOS score to at least 1). Importantly, oral intake can re-commence in up to 90% of patients. 

A review article by Jeurnink et al. [19] published in 2007 demonstrated results comparable with our study, with clinical success rates of 89%, early major complications of 7%, and a reintervention rate of 18% (mostly due to tumour ingrowth). A mean hospital stay of 7 days was quoted with mean survival of 105 days. In this meta-analysis, surgery was favoured for younger patients due to the higher rate of re-intervention in the endoscopic group. Based on our lower reintervention rates (5% versus 18%), this conclusion may not be so strongly indicated. 

The major shortcomings of all previous endoscopic studies have been the small numbers of patients involved. Our study reports on the largest cohort of patients undergoing gastroduodenal stenting for malignant obstruction examined to date. Furthermore, our study differentiated between patients according to the underlying malignancy and the location of obstruction, information which is lacking in many other studies. We demonstrated no significant difference in survival between gastric and pancreatic cancers, with a median of 127 days seen in each group. This is in contrast to 2006 report which showed that survival was shorter in stented patients with pancreatic cancer [20]. 

We note that stenting is not seen as effective in helping overcome obstruction from gastric cancer as opposed to pancreatic cancer often due to the location of the stenosis within the body of the stomach that does not allow for good expansion and often stent migration [21]. These factors may have led to a referral bias with less gastric cancers referred to our service for stenting. It was also seen that stenting in patients with gastric cancers had a decreased life expectancy. One possible explanation may be that these patients present later for intervention and hence portend a worse outcome.

In our study, we utilised two different stents from the same company (Boston) as these were the only two stents that were approved for the treatment of malignant gastroduodenal obstruction during the majority of our study period (up until 2011). All cases were undertaken by three experienced endoscopists (David Devonshire, Sina Alexander, and Michael P. Swan) with special interest and training in the insertion of gastroduodenal stents. 

Restenting occurred in 5 patients as a result of tumour ingrowth. This takes place through the wall of the stent and results in worsening gastroduodenal obstruction. All these patients all had successful reinsertion of stents and subsequent 1 month of increased life. Two of the patients with stent reinsertion had concurrent chemotherapy. The studies collecting data looking at concurrent systemic chemotherapy with stenting have not shown that chemotherapy in addition to stenting increases life expectancy [18, 20].

A potential weakness of our study is that patients were not prospectively randomised to either SEMs or surgery. However, a randomised study would be difficult to conduct as most patients with advanced disease are not surgical candidates. In fact, none of the patients in our cohort were deemed to be surgical candidates and many were referred to us by various surgical units. 

Examining the best quality published surgical data [13, 17], the clinical success rate for surgery in cases of malignant gastroduodenal obstruction is lower (72%) and is associated with a higher complication rate (33%). This is despite the fact that patients who are offered surgery are often younger [17], have early stage disease, and have less comorbidity. In nonrandomised trials, these same selection biases may also lead to a perception of improved survival for those undergoing surgery. Despite this, whilst our study supports the association of stent placement with more favorable short-term results, perhaps younger patients with increased likelihood of longer survival will be better served undergoing surgical bypass to avoid the potential need for reintervention. This may be especially relevant as chemotherapeutic modalities improve over time, improving patient survival rates.

## 5. Conclusion

This study reports on a large cohort of patients to demonstrate the clinical effectiveness of SEMs in achieving rapid symptomatic relief in patients with advanced gastroduodenal obstruction. When enteral stenting is undertaken by experienced endoscopists, SEMs have a high success rate with few complications, short hospitalisation and reduced need for re-interventions. 

## Figures and Tables

**Table 1:** Patient characteristics.

	Male	Female	Total
Number	43	51	94
Mean age (range) (years)	69 (28–92)	68 (40–93)	68 (28–93)
Tumour type			
Pancreatic			45 (48%)
Gastric			27 (29%)
Cholangio carcinoma/metastatic carcinoma			14 (16%)
Duodenal adenocarcinoma			8 (9%)

**Table 2:** Survival outcomes after stent insertion.

Type of tumour	Median survival (months)	Average (months)	Range (months)
Pancreatic *N* = 45	2	4.21	0.5–49
Gastric *N* = 27	2	3.53	0.5–49
Cholangiocarcinoma *N* = 12	2	4.18	0.5–10
Duodenal adenocarcinoma *N* = 8	2	4.25	0.15–30
Breast (metastatic) *N* = 2	3	6.28	0.25–9
